# Gestational weight gain rates in the first and second trimesters are associated with small for gestational age among underweight women: a prospective birth cohort study

**DOI:** 10.1186/s12884-022-04433-4

**Published:** 2022-02-05

**Authors:** Xueling Wei, Songying Shen, Peiyuan Huang, Xiong Xiao, Shanshan Lin, Lifang Zhang, Chengrui Wang, Min-Shan Lu, Jinhua Lu, Wing Hung Tam, Chi Chiu Wang, Jian-Rong He, Xiu Qiu

**Affiliations:** 1 Division of Birth Cohort Study, Guangzhou Women and Children’s Medical Center, Guangzhou Medical University, Guangzhou, 510623 China; 2 Department of Women’s Health, Provincial Key Clinical Specialty of Woman and Child Health, Guangzhou Women and Children’s Medical Center, Guangzhou Medical University, Guangzhou, 510623 China; 3 Provincial Clinical Research Center for Child Health, Guangzhou, 510623 China; 4 Department of Obstetrics and Gynaecology, The Chinese University of Hong Kong, Hong Kong, China; 5 Nuffield Department of Women’s and Reproductive Health, University of Oxford, Oxford, UK

**Keywords:** Gestational weight gain, Gestational weight gain rate, Small for gestational age, Underweight

## Abstract

**Background:**

Despite the well-studied effects of gestational weight gain (GWG) on offspring health, little is known about the association of trimester-specific GWG with offspring birth weight among underweight pregnant women. This study aimed to explore the association of trimester-specific GWG rate with small for gestational age (SGA) in underweight women.

**Methods:**

The GWG rate of underweight pregnant women (pre-pregnancy body mass index [BMI] lower than 18.5 kg/m^2^) of the Born in Guangzhou Cohort Study was calculated as the weight gain during a specific trimester divided by the corresponding duration of week. Total GWG was calculated as the weight difference between pre-pregnancy and delivery, and was categorized into inadequate, adequate, and excessive weight gain based on the 2009 Institute of Medicine (IOM) weight gain recommendation. The INTERGROWTH-21^st^ standards were used to define SGA. Logistic regression models were used to examine the associations of total GWG and trimester-specific GWG rates with SGA. Associations between trimester-specific GWG rates and SGA were also analyzed separately based on different total GWG categories (i.e. inadequate and adequate/excessive GWG).

**Results:**

Of the 3839 participants, SGA births occurred in 397 (10.3%), and mean GWG was 14.9 kg (SD 3.9). A lower risk of SGA was observed among women with higher GWG rate (per 0.5 kg/week increase) during the first (adjusted OR [aOR] 0.74, 95%CI 0.57, 0.96) and second (adjusted OR [aOR] 0.40, 95%CI 0.30, 0.55) but not third trimester. Similar association between higher GWG rate during the second trimester and a decreased risk of SGA were observed among women with inadequate (< 12.5 kg) and adequate/excessive (≥12.5 kg) total GWG, respectively. Compared to women with adequate GWG rate, women with inadequate GWG rate during the second trimester had a significantly increased risk of SGA (aOR 1.58, 95% CI 1.14, 2.20).

**Conclusions:**

Second-trimester GWG might be the key driver for the association between inadequate GWG and increased risk of SGA births in underweight women.

**Supplementary Information:**

The online version contains supplementary material available at 10.1186/s12884-022-04433-4.

## Introduction

Small for gestational age (SGA) is not only related to increased risks of neonatal and post-neonatal mortality [1], but also associated with neurodevelopmental problems and cardiometabolic diseases in later life [2]. In 2012, nearly one in five infants born with SGA in low and middle-income countries, associated with more than 20% of neonatal deaths [3]. China has the fifth highest number of SGA births all around the world, with a prevalence of 4.6% in 2012 [3]. Identification of modifiable risk factors is, therefore, of great importance for the prevention of SGA.

Gestational weight gain (GWG) is crucial for fetal growth and other perinatal outcomes [4]. Studies revealed that inadequate GWG is associated with an increased risk of SGA [4, 5]. The Institute of Medicine (IOM) guidelines recommended GWG range was based on pre-pregnancy body mass index (BMI). The optimal GWG recommendations are based on pre-pregnancy BMI categories: 12.5–18 kg for underweight women (BMI < 18.5 kg/m^2^); 11.5–16 kg for normal-weight women (BMI 18.5–24.9 kg/m^2^); 7–11.5 kg for overweight women (BMI 25–29.9 kg/m^2^); and 5–9 kg for women with obesity (BMI ≥30 kg/m^2^) [6]. However, adherence to these GWG recommendations is low. For example, in China, only about 40% of pregnant women had an adequate GWG recommended by the IOM [7, 8].

Specific GWG recommendations have also been proposed for women with different grades of obesity to provide more customized instructions [9, 10]. However, there is a lack of research focus on underweight women due to a relatively small proportion (1.8–5.7%) in western countries [4, 11, 12]. Instead, the prevalence of underweight women of reproductive age is as high as 14.2–21.7% in Asian countries (China, Japan, and Korea) [13–17]. Therefore, exploring the association of GWG with adverse pregnancy outcomes (such as SGA) among underweight women may have implications for GWG management among these populations.

In addition, GWG may have trimester-specific effects on fetal growth [6]. A recent study reported that GWG in the first two trimesters rather than the third trimester was positively related to offspring birth weight [18]. Another study argued that higher weight gain from mid-to late pregnancy led to higher birth weight [19]. Nevertheless, there is a lack of study investigating the associations between GWG rates during different trimesters and fetal growth among underweight women. Moreover, trimester-specific GWG may be more practical for clinical consultation and allow for timely intervention than total GWG which is unknown until delivery.

Given the high prevalence of underweight among Chinese pregnant women, it is important to clarify the impact of trimester-specific GWG rate on the risk of SGA in this population. This prospective cohort study aimed to explore the associations of total GWG and trimester-specific GWG rates with the risk of SGA among Chinese pregnant women who were underweight before pregnancy. SGA was selected as the only outcome in this study because it is the main concern for underweight or undernourished pregnant women in clinical practice. The findings of the present study can provide useful information on weight management to reduce SGA risk for underweight pregnant women in Asia.

## Methods

### Study design and participants

This study was based on the Born in Guangzhou Cohort Study (BIGCS), a prospective cohort study conducted in Guangzhou, China. The protocol of BIGCS was described in detail previously [20]. In brief, women who planned to give birth at the Guangzhou Women and Children’s Medical Center and live in Guangzhou for at least 3 years after delivery were invited to participate in the BIGCS at their first antenatal visit (< 20 weeks’ gestation). The study was approved by the Institutional Ethics Committee of the Guangzhou Women and Children’s Medical Center. Before recruitment, written informed consent was obtained from all participants.

Inclusion criteria for the current study were (1) pregnant women aged 18 years or above, (2) singleton pregnancies and (3) pre-pregnancy BMI < 18.5 kg/m^2^. The exclusion criteria were as follows: (1) withdrew before delivery, (2) diagnosed with diabetes, hypertension, and heart disease before pregnancy, (3) terminations of pregnancy or stillbirths, (4) multiple gestations or missing information on number of fetus, (5) preterm delivery, (6) missing weight data at delivery admission or implausible weight gain data.

### Exposure measurements

Measured height (in cm) and self-reported pre-pregnancy weight (in kg) were collected at recruitment by questionnaires and used to measure pre-pregnancy BMI (weight [kg]/height [m^2^]). Maternal weight during pregnancy was routinely measured multiple times (range 1–20, median 6) across pregnancy using the HGM-800A Height and Weight measuring scale at antenatal visits. The scale was calibrated every 6 months. Before the measurement, pregnant women were asked to take off heavy clothes, hat and shoes. All measurement results were recorded in obstetric notes. In this study, we extracted maternal weight data from obstetric records. During data cleaning, maternal weight was plotted against gestational age on a scatter plot. Outliers of maternal weight were then identified by visually screening the scatter plot and were removed before calculating GWG. Total GWG was calculated as the difference in maternal weight between pre-pregnancy and delivery. Based on the IOM recommendations for underweight women, total GWG was categorized into inadequate (< 12.5 kg), adequate (12.5–18 kg), and excessive GWG (> 18 kg) [6]. The time period of pregnancy was categorized as the first trimester (≤13^+ 6^ weeks), second trimester (14–27^+ 6^ weeks), and third trimester (≥28 weeks). GWG rate in the second and third trimesters was calculated as the difference between the earliest and the latest measurement of weight in the trimester divided by the number of weeks in the interval of the two measurements. When more than two measurements of a participant’s weight were made in a specific trimester, we selected the two measurements that were most distant in time. During the analysis, GWG rates during the second and third trimesters were categorized as inadequate (< 0.44 kg/week), adequate (0.44–0.58 kg/week) or excessive (> 0.58 kg/week) based on the recommendations for underweight women by the IOM. The absolute GWG amount in the second trimester was calculated by multiplying the GWG rate during the second trimester by 14, and the absolute GWG amount in the third trimester was calculated by multiplying the GWG rate during the third trimester by the number of gestational weeks in the third trimester (gestational age at birth minus 28). Because the absolute GWG amount in the first trimester was not directly measured, we calculated it by subtracting GWG in the second and third trimesters from total GWG. GWG rate during the first trimester was then estimated by dividing the absolute amount of GWG during the first trimester by 14 weeks. GWG rates during the first trimesters were categorized into three groups according to the IOM guidelines (assume a 0.5-2 kg weight gain in the first trimester). Groups of GWG rate during the first trimester were defined as follows: inadequate (< 0.04 kg/ week); adequate (0.04–0.15 kg/week); excessive (> 0.15 kg/week).

Incomplete data on maternal weight during the second trimester (*n* = 183) and the third trimester (*n* = 85) were imputed using linear interpolation from the R package called “zoo” [21]. For example, weight measurements at 12–13 gestational week and 15–16 gestational week were required for imputing weight at 14 weeks.

### Outcomes measurements

Data on birth characteristics, including birth weight (in g), infant sex, gestational age (in weeks), and delivery modes (vaginal delivery, cesarean section), were extracted from the hospital medical records. The birth weight Z score was calculated based on the INTERGROWTH-21^st^ standards [22]. The INTERGROWTH-21^st^ standards were derived from multiethnic populations (including Chinese) of healthy, well-nourished pregnant women and can be used to accurately assess the newborn size [22]. The INTERGROWTH-21^st^ study group has shown that the variations in fetal growth was mild among healthy pregnant women across different populations around the world [23]. Therefore, the INTERGROWTH-21^st^ standards were chosen as the standard to define SGA in our study. SGA, appropriate for gestational age (AGA) and large for gestational age (LGA), were defined as below 10th, from 10th to 90th, and above 90th percentiles of gestational age- and sex-specific birth weight according to the INTERGROWTH-21^st^ standards, respectively [22].

### Covariates

Self-administered questionnaires at recruitment were used to collect sociodemographic information, including maternal age (continuous), educational level (high school or below, vocational or technical college, undergraduate, postgraduate), parity (primipara, multipara), tobacco exposure during pregnancy (yes, no), folic acid supplementation during pregnancy (yes, no). Diagnosis of gestational diabetes mellitus was extracted from the hospital medical records.

### Statistical analyses

Continuous variables were described by the mean and standard deviation (SD), while categorical variables were reported as frequency and percentage. We first described the trajectory of maternal weight gain throughout pregnancy by total GWG categories (inadequate, adequate, and excessive GWG) and by neonatal size (SGA, AGA and LGA) using generalized additive model smoothing. General linear models were then used to evaluate the relationships of total GWG and trimester-specific GWG rates with birth weight Z score in two-step models. Model 1 was the unadjusted model. Model 2 was adjusted for maternal age (continuous), education level (ordinal: 1 for high school or below, 2 for vocational/technical college, 3 for undergraduate, and 4 for postgraduate), pre-pregnancy BMI (continuous), parity (binary:1 for multipara, and 0 for primipara), tobacco exposure (including active and passive smoking) during pregnancy (binary: 1 for yes, and 0 for no), gestational diabetes mellitus (binary: 1 for yes, and 0 for no), folic acid supplementation during pregnancy (binary: 1 for yes, and 0 for no), and weight gain during previous trimester. These covariates were selected based on a causal diagram (eFig. 1). Education level was included in the general linear models as a continuous variable, while binary covariates were included in the models as dummy variables. Logistic regression models were used to examine the association of total GWG and trimester-specific GWG rate with SGA, with the AGA category as the reference outcome, and adjusted for the confounders aforementioned. Both total GWG and GWG by trimester rate were analyzed as both continuous and categorical variables. We further conducted a sensitivity analysis to evaluate the extent to which unmeasured confounding may have played a role in shaping the observed associations based on E-values [24]. For an observed risk ratio of OR: E-value = OR + sqrt {OR×(OR-1)}. The formula was applied to an OR greater than 1; for an OR less than 1, we first took the inverse of the observed OR and then applied the formula. For categorical variables analysis, women within adequate total GWG or adequate GWG rate during each trimester were used as the reference group. Further, a stratified analysis was performed for the associations between GWG rates and birth weight and the risk of SGA by total GWG categories (inadequate, adequate, and excessive).

All analyses were performed using R version 3.6.1 or SAS statistical software version 9.2 (SAS Institute Inc., Cary, NC, USA). A two-tailed *p*-value< 0.05 was considered statistically significant.

## Results

**Fig. 1:**
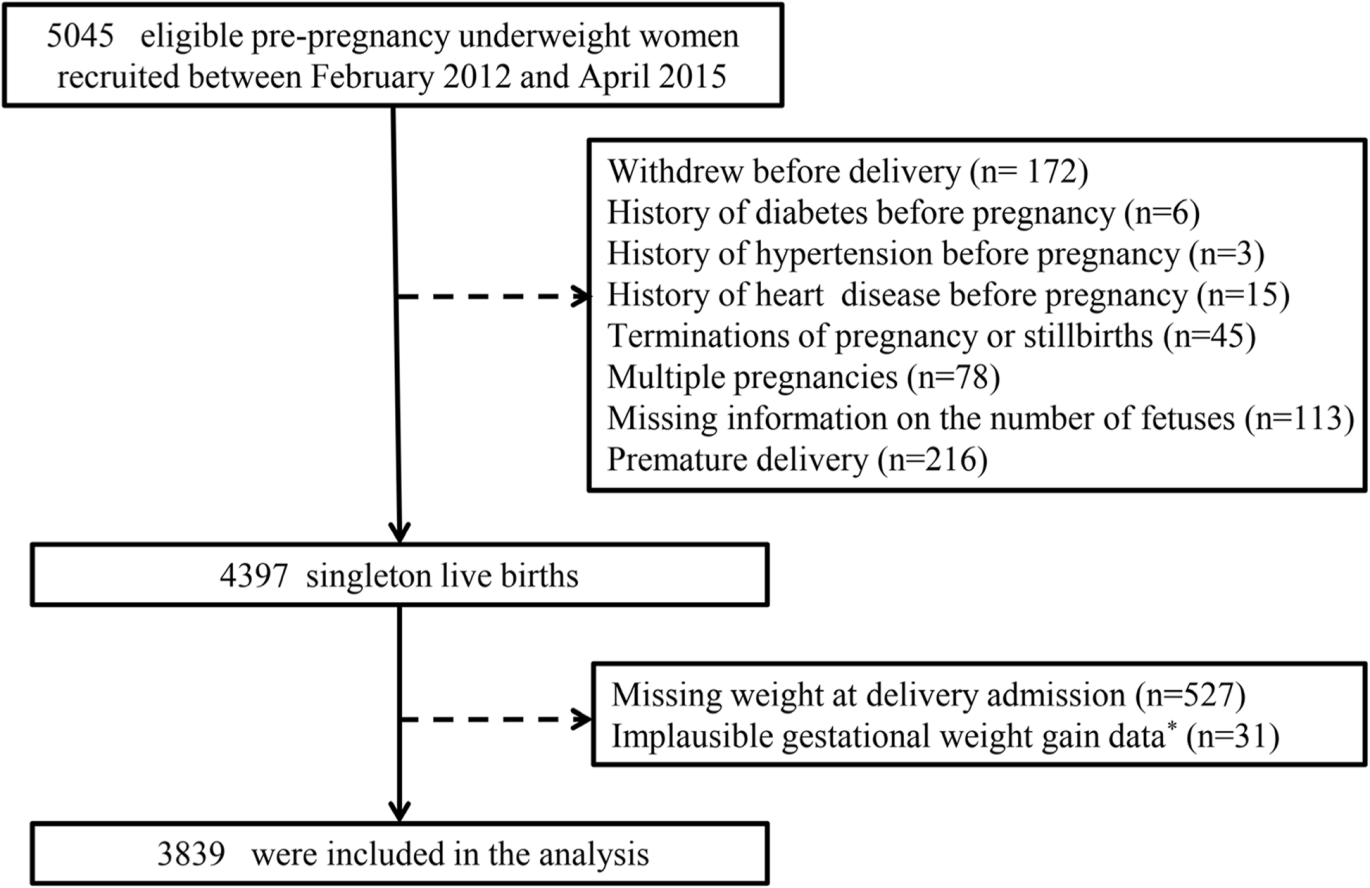
Selection process of study population in the Born in Guangzhou Cohort Study (BIGCS). *Defined as 3 standard deviations above or below the mean total gestational weight gain

There were 22,081 women recruited between February 2012 and April 2015 in the BIGCS, of which 5045 (22.8%) were underweight before pregnancy and eligible for the current study. 1206 (23.9%) were excluded based on the exclusion criteria, resulting in 3839 participants for analysis (Fig. 1).

**Fig. 2:**
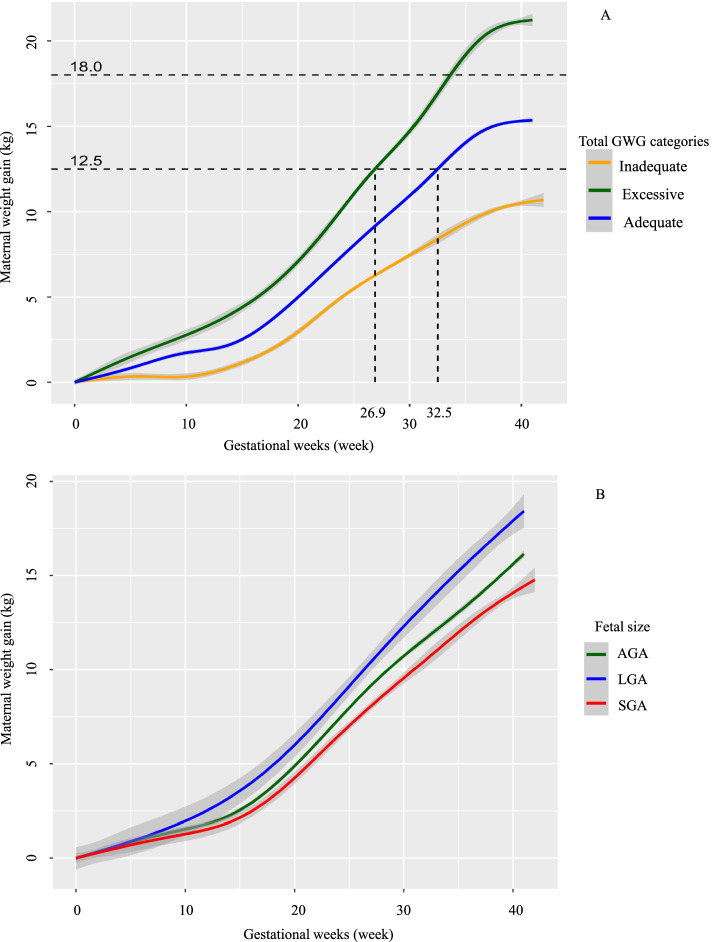
Maternal weight gain throughout pregnancy by total GWG categories and fetal size among underweight women. **A**: Maternal weight gain throughout pregnancy by total GWG categories; **B**: Maternal weight gain throughout pregnancy by fetal size. The curves (95%CI, indicated by light grey shading) were derived from ggplot2 smoothing plots (PROC GAM). SGA, small for gestational age; AGA, appropriate for gestational age; LGA, large for gestational age. GWG, gestational weight gain

Table 1 described the demographic and clinical details of SGA, AGA, LGA in pre-pregnancy underweight women in the study. Of the 3839 women included, 397 (10.3%) had SGA, 3342 (87.1%) had AGA, and 100 (2.6%) had LGA babies. Over 50% of the mothers had education at the undergraduate level, and over 85% of mothers took folic acid supplementation during pregnancy. Mean GWG among mothers with SGA, AGA, LGA babies were 13.7 kg (SD 3.7), 14.9 kg (SD 3.9), and 17.3 kg (SD 5.1), respectively. Maternal weight gain throughout pregnancy by total GWG categories and fetal size was presented in Fig. 2. Compared to women with inadequate GWG, women with adequate GWG and excessive GWG gained 12.5 kg in the 32nd and the 26th week of gestation, respectively. Women with SGA babies gained the least weight during the whole gestation, compared to the women with AGA or LGA babies.

**Table 1:** Characteristics of pre-pregnancy underweight women

Characteristics	Total (***n*** = 3839)	Fetal size		
		SGA (***n*** = 397)	AGA (***n*** = 3342)	LGA (***n*** = 100)
**Maternal Characteristics**				
Maternal age, year, mean (SD)	28.5 (3.3)	28.3 (3.3)	28.5 (3.3)	29.7 (3.9)
Educational level, n (%)				
High school or below	371 (9.7)	38 (9.6)	326 (9.8)	7 (7.0)
Vocational/technical college	1027 (26.8)	124 (31.2)	885 (26.5)	18 (18.0)
Undergraduate	2094 (54.5)	216 (54.4)	1825 (54.6)	53 (53.0)
Postgraduate	347 (9.0)	19 (4.8)	306 (9.2)	22 (22.0)
Monthly income (Yuan), n (%)				
≤ 1500	410 (11.1)	35 (9.2)	368 (11.4)	7 (7.5)
1501–4500	1157 (31.3)	137 (35.9)	996 (31.0)	24 (25.8)
4501–9000	1483 (40.2)	159 (41.6)	1289 (40.1)	35 (37.6)
≥ 9001	642 (17.4)	51 (13.4)	564 (17.5)	27 (29.0)
Tobacco exposure during pregnancy, n (%)	1677 (43.7)	192 (48.4)	1445 (43.2)	40 (40.0)
Folic acid supplementation during pregnancy, n (%)	3343 (87.6)	342 (86.6)	2910 (87.6)	91 (91.0)
Primipara, n (%)	3278 (85.4)	358 (90.2)	2841 (85.0)	79 (79.0)
Pre-pregnancy BMI, kg/m^2^, mean (SD)	17.5 (0.8)	17.4 (0.9)	17.5 (0.8)	17.5 (1.0)
Gestational weight gain, kg, mean (SD)	14.9 (3.9)	13.7 (3.7)	14.9 (3.9)	17.3 (5.1)
IOM weight gain category across gestation				
Inadequate (< 12.5 kg)	989 (25.8)	141 (35.5)	830 (24.8)	18 (18.0)
Adequate (12.5–18.0 kg)	2176 (56.7)	219 (55.2)	1913 (57.2)	44 (44.0)
Excessive (> 18.0 kg)	674 (17.6)	37 (9.3)	599 (17.9)	38 (38.0)
Gestational weight gain rate, g/week, mean (SD)				
First trimester	159.2 (254.7)	126.3 (236.3)	162.2 (256.3)	201.3 (267.0)
Second trimester	590.3 (227.7)	536.4 (207.4)	594.4 (228.9)	683.8 (228.9)
Third trimester	471.7 (220.1)	461.2 (241.8)	471.4 (215.2)	530.9 (283.2)
**Offspring Characteristics**				
Male, n (%)	1970 (51.3)	199 (50.1)	1721 (51.5)	50 (50.0)
Birthweight, g, mean (SD)	3132.1 (351.5)	2603.8 (210.5)	3171.4 (286.6)	3916.9 (216.7)
Low birthweight (< 2500 g), n (%)	105 (2.7)	97 (24.4)	8 (0.2)	0 (0.0)
Gestational age, median (25th,75th percentile)	39 (38, 40)	39 (38, 40)	39 (38, 40)	39 (38, 40)
Vaginal delivery, n (%)	2864 (74.6)	322 (81.1)	2491 (74.6)	51 (51.0)

*SGA* small for gestational age, *AGA* appropriate for gestational age, *LGA* large for gestational age, *IOM* the Institute of Medicine, now known as the National Academy of Medicine

**Fig. 3:**
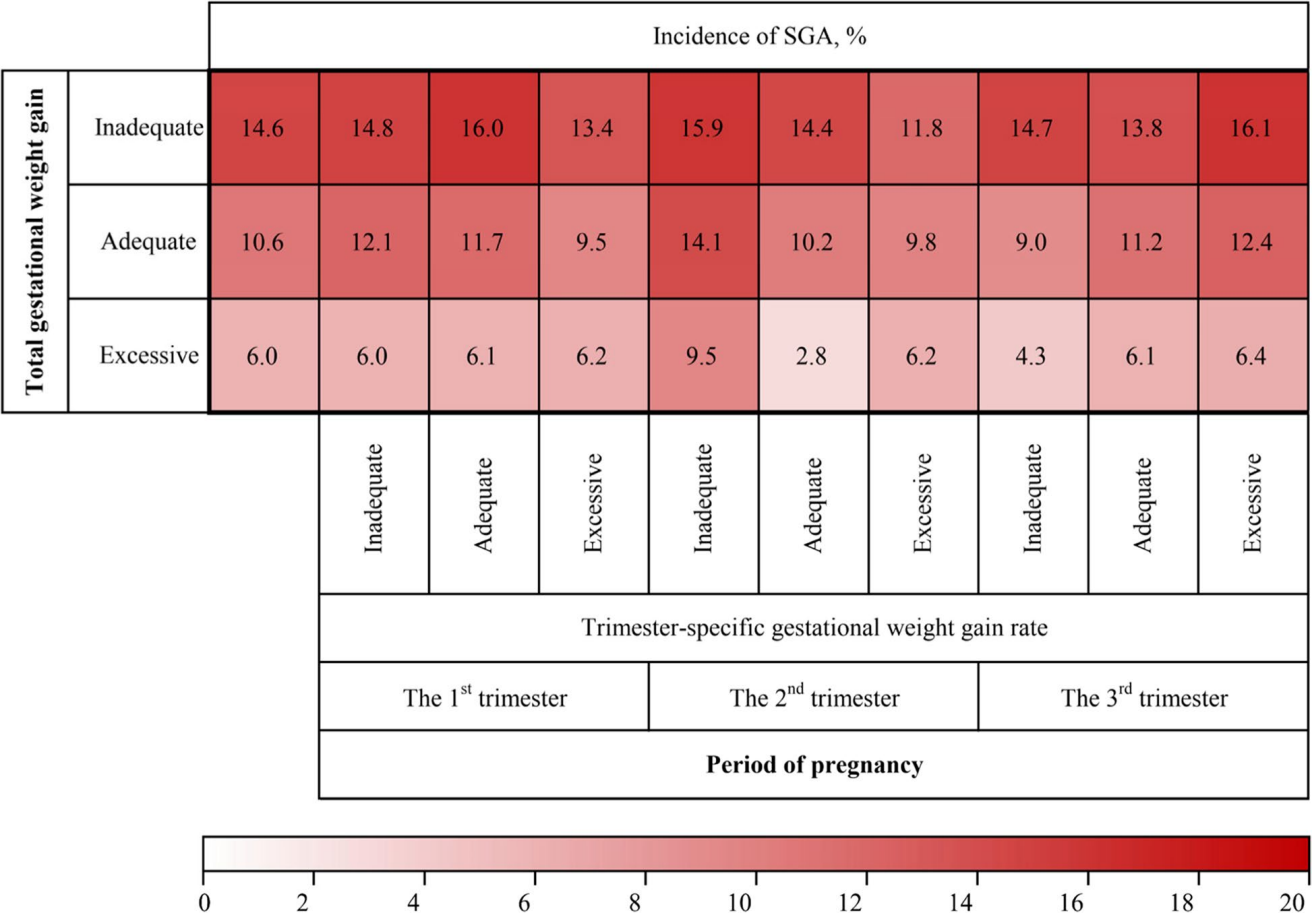
Incidence of SGA by total GWG and trimester-specific GWG rate among underweight women. SGA, small for gestational age; GWG, gestational weight gain

Incidence of SGA by total GWG and trimester-specific GWG rate among underweight women was shown in Fig. 3. The incidence of SGA for women with adequate total GWG and inadequate GWG rate during the second trimester was 14.1%. Associations between GWG and birth weight were shown in Table 2. In this study, increasing trends were observed between total GWG, trimester-specific GWG rate, and birth weight Z scores, respectively. The positive association between total GWG, trimester-specific GWG rate, and birth weight Z scores persisted even after the adjustment for potential confounders. As a continuous variable (per 1 kg increase), total GWG was inversely associated with SGA (adjusted OR [aOR] 0.91, 95% CI 0.89, 0.94). Similar associations were observed between the GWG rate (per 0.5 kg/week increase) during both the first (aOR 0.74, 95% CI 0.57, 0.96) and second trimester (aOR 0.40, 95% CI 0.30, 0.55) with the risk of SGA. For categorical GWG, inadequate GWG was associated with increased risk of SGA (aOR 1.52, 95% CI 1.19, 1.93), while excessive GWG was related to a decreased risk of SGA (aOR 0.54, 95% CI 0.37, 0.78), compared to the group with adequate total GWG. For categorical GWG rate during the first trimester, women with inadequate GWG rate were not associated with SGA, but excessive GWG rate was associated with a decreased risk of SGA (aOR 0.70, 95% CI 0.51, 0.96), as compared with adequate GWG rate. For categorical GWG rate during the second trimester, women with inadequate GWG rate (aOR 1.58, 95% CI 1.14, 2.20) had a significantly increased risk of SGA as compared with adequate GWG rate. For third trimesters, no significant difference in the odds of SGA was observed among subjects with excessive or inadequate GWG rates when compared with adequate GWG rate.

**Table 2:** Association between gestational weight gain (GWG) and GWG rate with small for gestational age (SGA) among pre-pregnancy underweight women

GWG	Birthweight Z-scores			SGA ^**a**^		
	N (%)	Crude ***β*** (95%CI)	Adjusted ***β*** (95%CI) ^**b**^	N (%)	Crude OR (95%CI)	Adjusted OR (95%CI) ^**b**^
Total GWG						
Continuous (per 1 kg increase)	3839	**0.04 (0.04, 0.05)**	**0.05 (0.04, 0.05)**	397	**0.91 (0.89, 0.94)**	**0.91 (0.89, 0.94)**
Inadequate (< 12.5 kg)	989 (25.8)	**− 0.20 (− 0.26, − 0.14)**	**−0.20 (− 0.27, − 0.14)**	141 (14.5)	**1.48 (1.18, 1.86)**	**1.52 (1.19, 1.93)**
Adequate (12.5-18 kg)	2176 (56.7)	0.00 (Reference)	0.00 (Reference)	219 (10.3)	1.00 (Reference)	1.00 (Reference)
Excessive (> 18.0 kg)	674 (17.6)	**0.28 (0.21, 0.35)**	**0.30 (0.23, 0.37)**	37 (5.8)	**0.54 (0.38, 0.77)**	**0.54 (0.37, 0.78)**
GWG rate in the first trimester						
Continuous (per 0.5 kg/week increase)	2861	**0.18 (0.12, 0.24)**	**0.18 (0.11, 0.24)**	307	**0.74 (0.58, 0.95)**	**0.74 (0.57, 0.96)**
Inadequate (< 0.04 kg/week)	862 (30.1)	−0.03 (−0.12, 0.05)	−0.02 (− 0.11, 0.06)	106 (12.5)	1.02 (0.74, 1.39)	0.98 (0.71, 1.35)
Adequate (0.04–0.15 kg/week)	635 (22.2)	0.00 (Reference)	0.00 (Reference)	77 (12.4)	1.00 (Reference)	1.00 (Reference)
Excessive (> 0.15 kg/week)	1364 (47.7)	**0.11 (0.04, 0.19)**	**0.13 (0.05, 0.20)**	124 (9.3)	**0.73 (0.54, 0.98)**	**0.70 (0.51, 0.96)**
GWG rate in the second trimester						
Continuous (per 0.5 kg/week increase)	2889	**0.21 (0.14, 0.27)**	**0.34 (0.27, 0.41)**	308	**0.56 (0.42, 0.73)**	**0.40 (0.30, 0.55)**
Inadequate (< 0.44 kg/week)	703 (24.3)	**−0.09 (−0.17, −0.01)**	**−0.14 (− 0.22, − 0.05)**	102 (14.7)	**1.44 (1.06, 1.97)**	**1.58 (1.14, 2.20)**
Adequate (0.44–0.58 kg/week)	779 (27.0)	0.00 (Reference)	0.00 (Reference)	82 (10.7)	1.00 (Reference)	1.00 (Reference)
Excessive (> 0.58 kg/week)	1407 (48.7)	**0.13 (0.06, 0.20)**	**0.20 (0.13, 0.27)**	124 (9.1)	0.84 (0.62, 1.12)	**0.70 (0.51, 0.95)**
GWG rate in the third trimester						
Continuous (per 0.5 kg/week increase)	3003	0.06 (−0.01, 0.13)	0.06 (− 0.01, 0.13)	319	0.90 (0.69, 1.17)	0.93 (0.70, 1.24)
Inadequate (< 0.44 kg/week)	1307 (43.5)	< 0.01 (−0.07, 0.07)	−0.04 (− 0.12, 0.03)	143 (11.1)	1.01 (0.77, 1.34)	1.13 (0.84, 1.53)
Adequate (0.44–0.58 kg/week)	842 (28.0)	0.00 (Reference)	0.00 (Reference)	91 (11.0)	1.00 (Reference)	1.00 (Reference)
Excessive (> 0.58 kg/week)	854 (28.4)	0.06 (−0.02, 0.14)	0.02 (−0.06, 0.10)	85 (10.4)	0.94 (0.69, 1.28)	1.07 (0.77, 1.49)

^a^ The reference group was those who had appropriate for gestational age infants

^b^ The multivariable models were adjusted for maternal age, education level, pre-pregnancy BMI, parity, tobacco exposure during pregnancy, gestational diabetes mellitus, and folic acid supplementation during pregnancy. GWG rate during the second trimester models were further adjusted for GWG rate during the first trimester (categorical variable: −1, 0, 1. The values of GWG rate during the first trimester were defined based on the IOM recommends: − 1, < 0.04 kg/ week; 0, 0.04–0.15 kg/week; 1, > 0.15 kg/week). GWG rate during the third trimester models are further adjusted for GWG rate during the first (categorical variable as aforementioned) and the second trimester (categorical variable: − 1, 0, 1. The values of GWG rate during the second trimester were defined based on the IOM recommends: − 1, < 0.44 kg/week; 0, 0.44–0.58 g/week; 1, > 0.58 g/week)

The values highlighted in bold are statistically significant (*p* < 0.05)

The results of stratified analyses by total GWG categories were showed in eTable 2. Among women with inadequate total GWG throughout pregnancy (< 12.5 kg), GWG rate during the second trimester (per 0.5 kg/week increase) was related to increased birth weight Z scores (adjusted *β* 0.23, 95% CI 0.05, 0.42) and a decreased risk of SGA (aOR 0.40, 95% CI 0.19, 0.84). For women with adequate or excessive GWG (≥12.5 kg), GWG rate in the second trimester (adjusted *β* 0.30, 95% CI 0.21, 0.39) was similarly associated with increased birth weight Z-score and a reduced risk of SGA (aOR 0.41, 95% CI 0.28, 0.62). However, no significant associations between SGA and GWG rate during the first or third trimester was observed. The E-values for observed OR varied from 1.31 to 4.70 for the association of trimester-specific GWG rate and SGA, which indicated considerable unmeasured confounding would be needed to explain away these associations.

## Discussion

This is a longitudinal study of trimester-specific GWG rates and SGA among underweight women in a population with a high proportion (22.8%) of underweight. In this study, total GWG was positively related to birth weight and negatively associated with the risk of SGA. For the trimester-specific GWG rates, we found GWG rates in the first and second trimesters, but not in the third trimester, were associated with birth weight or SGA, with strong association for GWG rate in the second trimester.

Our findings on the association of total GWG and SGA were consistent with a large number of previous studies, which have found that total GWG below the IOM guidelines was associated with a higher risk of SGA. A recent meta-analysis of 23 studies concluded that total GWG below the recommendations increased the risk of SGA (aOR 1.53, 95% CI 1.44–1.64), and that the association was most pronounced in pre-pregnancy underweight women (aOR 1.89, 95% CI 1.67–2.14) [4].

Our findings add new evidence for the effects of trimester-specific GWG rate on SGA among underweight women. This result is consistent with a retrospective analysis performed in 472 pregnant women which shows an increase in GWG rates in the first and second trimesters were associated with lower risk of SGA [25]. GWG rate in the second trimester instead of the third trimester was the main driver of maternal weight gain for the birth weight [25]. The Agency for Healthcare Research and Quality’s (AHRQ) review also showed the increase in GWG per unit during the first or second trimester has a stronger effect on birth weight than that during the third trimester [26]. In a recent study conducted among rural nulliparous, women with inadequate weight gain rate from mid-to late pregnancy had a higher increased risk of SGA compared to those meeting the recommendations based on the 2009 IOM guidelines [27]. A retrospective cohort study from in upstate New York including white (73.7%), African (14.3%) and Asian (9.3%), and other races suggested that the women with a less-than-recommended GWG rate (< 0.44 kg/week) in the second and third trimesters were related to an increased risk of SGA among underweight women [28]. However, the limited number of time points for weight data collection, previous studies could not differentiate the effects of GWG rate between the second and third trimester on SGA [25, 27, 28].

We also found that excessive GWG rate during both the first and second trimester were related to a decreased risk of SGA, compared to the group with adequate GWG rate. These findings suggest that the optimal range of GWG among underweight pregnant women to reduce SGA risk could be wider than the current recommendations. Given the high proportion of underweight women in China, Japan, Senegal, and other part of Asia and Africa [29], our findings could have significant implications for the importance of regular weight monitoring and timely weight management during pregnancy.

We found that a low GWG rate in the second trimester is more detrimental to the risk of SGA than that in the first and third trimesters. Similar findings were observed in the stratified analysis by whether the women gained adequate weight based on the IOM recommended or not. This suggests that the second trimester might be the sensitive period of GWG for birth weight and could be the intervention window to prevent SGA in underweight women.

There are several potential pathways that could explain the associations we observed. For example, GWG may influence glucose and insulin regulation and metabolism in the fetus. It has been reported that greater GWG rate in first trimester was associated with higher insulin and lower adiponectin levels in cord blood, whereas greater second trimester GWG rate was associated with higher cord blood levels of insulin-like growth factors (IGF) and leptin [30]. These metabolic factors are well-known determinants for fetal growth. The second trimester is an essential period for the development of adipose tissue and fetal organs [31]. GWG rate during the second trimester primarily represents the growth of maternal plasma volume and fat deposition, which may increase the placental transfer of nutrients from mother to fetus and relate to the increase of fetal size [6, 32]. Low GWG rate in this period may cause placenta bloodstream perfusion insufficiency and cause fetal growth restriction, in turn leading to SGA. Excessive GWG rate during the second trimester may reflect exposure of the fetus to greater amounts of glucose and fatty acids during growth, which may result in decreased risk of SGA. On the other hand, it was hypothesized that GWG in the third-trimester may influence fetal body composition [33, 34] more than weight. Therefore, there may be a critical window may exist for the effect of GWG on fetal growth, though the underlying mechanisms merit further exploration.

The strengths of our study include the prospective design and multiple antenatal weight gain measurements which allow us to assess trimester-specific GWG rate. In addition, the relatively high proportion of underweight women in China allows us to conduct this research to focus on this less studied population. Some limitations should be acknowledged. First, self-reported pre-pregnancy weight was used, which could not exclude the possibility of misclassification error of the exposure. However, high correlations between self-reported and measured pre-pregnancy weights have been reported in many previous studies [35, 36]. Second, some potential confounding factors, including physical activity intensity and diet data, were not included in the analysis. Sensitive analysis using E-values declared that relatively influential unmeasured confounders would be needed to negate the observed associations, for example, an unmeasured confounder associated with inadequate GWG rate during the second trimester and SGA by an OR of 2.77 to remove the observed association toward the null. Third, our study only included a Chinese population, which may limit the generalizability of our results to other populations. Fourth, we excluded 558 eligible mother-child pairs due to missing weight at delivery admission or implausible GWG data. However, there were only minor differences in maternal and birth characteristics between births with and without GWG information for analyses (eTable 1). Finally, the observational nature of this study cannot exclude residual confounding, thus limiting causal inference.

## Conclusions

This prospective cohort study shows that increased GWG rates in the first and second trimesters, but not in the third trimester, were associated with lower risk of SGA among pregnant women who were underweight before pregnancy. The associations for GWG rate in the second trimester were stronger than that in the first trimester and were independent of the total GWG amount. These findings suggest that the first and second trimesters, especially the second trimester, might be critical periods for GWG affects fetal growth. Therefore, monitoring weight gain and administering timely intervention during these periods may help reduce the risk of SGA in pre-pregnancy underweight women. Future studies are needed to confirm our findings and develop strategies of GWG management for pre-pregnancy underweight women.

## Supplementary Information

**Additional file 1: eTable 1**. Characteristics of births with and without GWG information for analyses. **eTable 2**. Association between GWG rate and birth weight among pre-pregnancy underweight women with inadequate, adequate and excessive GWG. **eFigure 1**. Directed acyclic graph representing potential confounders and mediators on the association between trimester-specific GWG rate and small for gestational age.

## Data Availability

The data that support the findings of this study are available from the Guangzhou Women and Children’s Medical Center but restrictions apply to the availability of these data, which were used under license for the current study, and so are not publicly available. Data are however available from the authors upon reasonable request and with permission of Guangzhou Women and Children’s Medical Center and relevant administrative departments.

## Abbreviations

GWG: Gestational weight gain
SGA: Small for gestational age
95% CI: 95% confidence interval
OR: Odds Ratio
aOR: Adjusted Odds Ratio
IOM: The Institute of Medicine
BIGCS: Born in Guangzhou Cohort Study
BMI: Body mass index
