# Supplementary material for: Gestational weight gain rates in the first and second trimesters are associated with small for gestational age among underweight women: a prospective birth cohort study
Source: BMC Pregnancy Childbirth. 2022 Feb 5;22:106. doi: 10.1186/s12884-022-04433-4 (PMC8818159; doi:10.1186/s12884-022-04433-4)
Supplement: Supplementary file 1 — Additional file 1: eTable 1. Characteristics of births with and without GWG information for analyses. eTable 2. Association between GWG rate and birth weight among pre-pregnancy underweight women with inadequate, adequate and excessive GWG. eFigure 1. Directed acyclic graph representing potential confounders and mediators on the association between trimester-specific GWG rate and small for gestational age. [file 12884_2022_4433_MOESM1_ESM.doc]

| eTable 1. Characteristics of births with and without gestational weight gain information for analyses. | | |
| --- | --- | --- |
| Characteristics | With information (N=3839), N (%) | Missing information (N=558), N (%) |
| Maternal Characteristics |  |  |
| Maternal age, year, mean (SD) | 28.5 (3.3) | 28.4 (3.3) |
| Educational level, n (%) |  |  |
| High school or below | 372 (9.7) | 96 (17.2) |
| Vocational/technical college | 1027 (26.8) | 153 (27.4) |
| Undergraduate | 2093 (54.5) | 254 (45.5) |
| Postgraduate | 347 (9.0) | 55 (9.9) |
| Monthly income (Yuan), n (%) |  |  |
| ≤1500 | 410 (11.1) | 77 (14.6) |
| 1501-4500 | 1157 (31.3) | 182 (34.5) |
| 4501-9000 | 1483 (40.2) | 190 (36.0) |
| ≥9001 | 642 (17.4) | 79 (15.0) |
| Tobacco exposure during pregnancy, n (%) | 1677 (47.3) | 239 (56.5) |
| Folic acid supplementation during pregnancy, n (%) | 3343 (87.6) | 479 (88.1) |
| Primipara, n (%) | 3278 (85.4) | 468 (83.9) |
| Pre-pregnancy BMI, kg/m^2^, mean (SD) | 17.5 (0.8) | 17.4 (0.9) |
| Offspring Characteristics |  |  |
| Male, n (%) | 1970 (51.3) | 294 (52.7) |
| Birthweight, g, mean (SD) | 3132.1 (351.5) | 3117.3 (370.6) |
| Gestational age, median (25^th^ ,75^th^ percentile) | 39 (38, 40) | 39 (38, 40) |
| Vaginal delivery, n (%) | 973 (25.4) | 150 (27.2) |

| **eTable 2.** Association between GWG rate and birth weight among pre-pregnancy underweight women with inadequate, adequate and excessive GWG. | | | | |
| --- | --- | --- | --- | --- |
| GWG | N | Birthweight Z-scores |  | SGA |
|  |  | Adjusted β (95%CI) ^b^ |  | Adjusted OR (95%CI) ^b^ |
| Restricted to women with inadequate GWG (<12.5kg) | | | | |
| GWG rate in the first trimester |  |  |  |  |
| Continuous ^c^ | 725 | 0.07 (-0.07, 0.20) |  | 0.99 (0.58, 1.69) |
| Inadequate (<0.04kg/week) | 328 | -0.04 (-0.18, 0.11) |  | 0.78 (0.46, 1.33) |
| Adequate (0.04-0.15kg/week) | 183 | Reference |  | Reference |
| Excessive (>0.15kg/week) | 214 | 0.01 (-0.15, 0.17) |  | 0.73 (0.40, 1.32) |
| GWG rate in the second trimester |  |  |  |  |
| Continuous ^c^ | 742 | **0.23 (0.05, 0.42)** |  | **0.40 (0.19, 0.84)** |
| Inadequate (<0.44kg/week) | 350 | -0.08 (-0.23, 0.06) |  | 1.27 (0.74, 2.16) |
| Adequate (0.44-0.58kg/week) | 218 | Reference |  | Reference |
| Excessive (>0.58kg/week) | 174 | **0.17 (0.01, 0.34)** |  | 0.62 (0.32, 1.19) |
| GWG rate in the third trimester |  |  |  |  |
| Continuous ^c^ | 757 | 0.04 (-0.13, 0.21) |  | 0.66 (0.35, 1.25) |
| Inadequate (<0.44kg/week) | 537 | -0.10 (-0.24, 0.05) |  | 1.31 (0.74, 2.31) |
| Adequate (0.44-0.58kg/week) | 161 | Reference |  | Reference |
| Excessive (>0.58kg/week) | 59 | -0.19 (-0.44, 0.06) |  | 1.21 (0.48, 3.05) |
| Restricted to women with adequate or excessive GWG (≥12.5kg) | | | | |
| GWG rate in the first trimester |  |  |  |  |
| Continuous ^c^ | 2136 | **0.14 (0.07, 0.22)** |  | 0.78 (0.57, 1.07) |
| Inadequate (<0.04kg/week) | 534 | 0.01 (-0.09, 0.12) |  | 1.04 (0.69, 1.58) |
| Adequate (0.04-0.15kg/week) | 452 | Reference |  | Reference |
| Excessive (>0.15kg/week) | 1150 | **0.12 (0.03, 0.21)** |  | 0.76 (0.52, 1.10) |
| GWG rate in the second trimester |  |  |  |  |
| Continuous ^c^ | 2147 | **0.30 (0.21, 0.39)** |  | **0.41 (0.28, 0.62)** |
| Inadequate (<0.44kg/week) | 353 | **-0.11 (-0.22, <0.001) ^d^** |  | **1.75 (1.12, 2.72)** |
| Adequate (0.44-0.58kg/week) | 561 | Reference |  | Reference |
| Excessive (>0.58kg/week) | 1233 | **0.17 (0.09, 0.26)** |  | 0.77 (0.53, 1.13) |
| GWG rate in the third trimester |  |  |  |  |
| Continuous ^c^ | 2246 | 0.01 (-0.08, 0.10) |  | 1.12 (0.77, 1.63) |
| Inadequate (<0.44kg/week) | 770 | 0.01 (-0.08, 0.10) |  | 1.01 (0.69, 1.49) |
| Adequate (0.44-0.58kg/week) | 681 | Reference |  | Reference |
| Excessive (>0.58kg/week) | 795 | 0.03 (-0.06, 0.12) |  | 1.04 (0.72, 1.51) |
| a. The reference group was those who had appropriate for gestational age infants.  b. The multivariable models were adjusted for maternal age, education level, pre-pregnancy BMI, parity, tobacco exposure during pregnancy, gestational diabetes mellitus, and folic acid supplementation during pregnancy. GWG rate during the second trimester models were further adjusted for GWG rate during the first trimester (categorical variable: -1, 0, 1. The values of GWG rate during the first trimester were defined based on the IOM recommends: -1, <0.04kg/ week; 0, 0.04-0.15kg/week; 1, >0.15kg/week). GWG rate during the third trimester models were further adjusted for GWG rate during the first (categorical variable as aforementioned) and the second trimester (categorical variable: -1, 0, 1. The values of GWG rate during the second trimester were defined based on the IOM recommends: -1, <0.44kg/week; 0, 0.44-0.58g/week; 1, >0.58g/week).  c. For per 0.5 kg/week increase.  d. P=0.051.  The values highlighted in bold are statistically significant (p<0.05). | | | | |


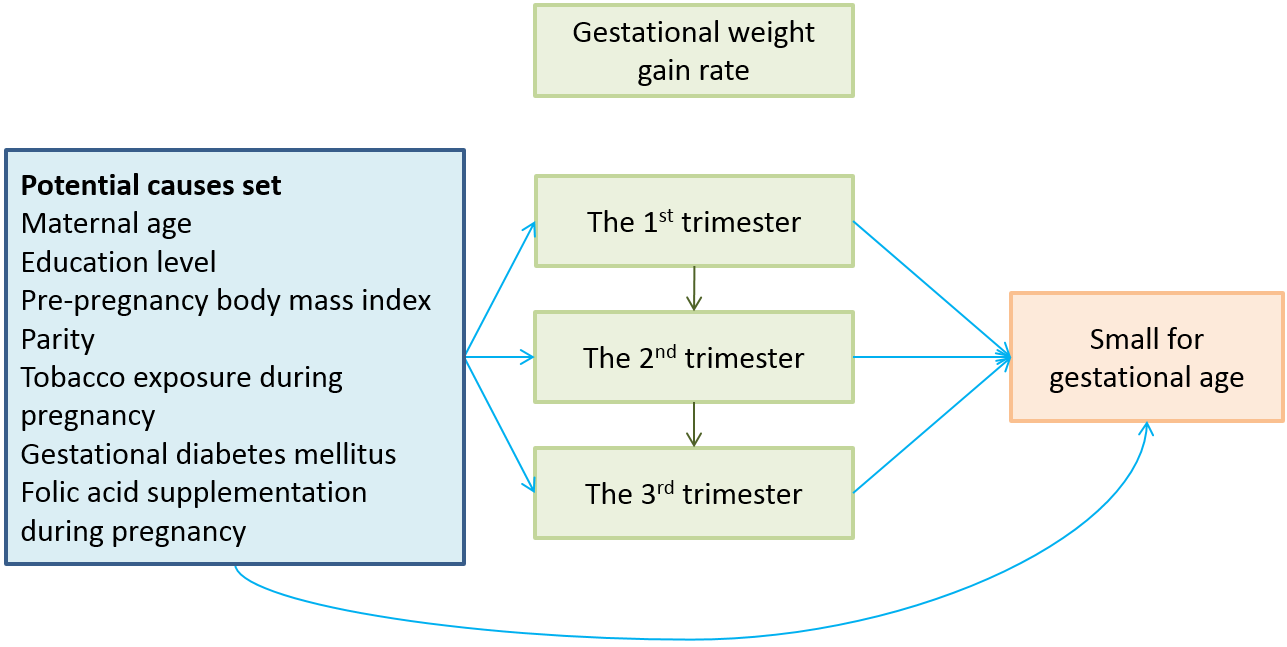


**eFigure 1.** Directed acyclic graph representing potential confounders and mediators on the association between trimester-specific gestational weight gain rate and small for gestational age
